# *In vitro* skin models for pigmentary disorders: from reconstructed tissues to skin-on-a-chip platforms

**DOI:** 10.1093/rb/rbag165

**Published:** 2026-08-05

**Authors:** Tianxiao Wei, Zehui Li, Zilin Zhang, Jing Zhang, Jun Ouyang, Nuo Si, Zhongze Gu, Zaozao Chen, Ningbei Yin

**Affiliations:** Plastic Surgery Hospital, Chinese Academy of Medical Sciences and Peking Union Medical College, Beijing 100144, China; Skin-on-a-Chip Translational Medicine Center, Joint Laboratory of Plastic Surgery Hospital, Chinese Academy of Medical Sciences and Southeast University, Beijing 100144, China; Plastic Surgery Hospital, Chinese Academy of Medical Sciences and Peking Union Medical College, Beijing 100144, China; Skin-on-a-Chip Translational Medicine Center, Joint Laboratory of Plastic Surgery Hospital, Chinese Academy of Medical Sciences and Southeast University, Beijing 100144, China; State Key Laboratory of Digital Medical Engineering, Institute of Microphysiological Systems, School of Biological Science and Medical Engineering, Southeast University, Nanjing 210096, China; Skin-on-a-Chip Translational Medicine Center, Joint Laboratory of Plastic Surgery Hospital, Chinese Academy of Medical Sciences and Southeast University, Beijing 100144, China; State Key Laboratory of Digital Medical Engineering, Institute of Microphysiological Systems, School of Biological Science and Medical Engineering, Southeast University, Nanjing 210096, China; Laboratory of Organs-on-a-Chip Research, Jiangsu Avatarget Biotechnology Co., Ltd, Suzhou 215163, China; Skin-on-a-Chip Translational Medicine Center, Joint Laboratory of Plastic Surgery Hospital, Chinese Academy of Medical Sciences and Southeast University, Beijing 100144, China; Laboratory of Organs-on-a-Chip Research, Jiangsu Avatarget Biotechnology Co., Ltd, Suzhou 215163, China; Plastic Surgery Hospital, Chinese Academy of Medical Sciences and Peking Union Medical College, Beijing 100144, China; Skin-on-a-Chip Translational Medicine Center, Joint Laboratory of Plastic Surgery Hospital, Chinese Academy of Medical Sciences and Southeast University, Beijing 100144, China; State Key Laboratory of Digital Medical Engineering, Institute of Microphysiological Systems, School of Biological Science and Medical Engineering, Southeast University, Nanjing 210096, China; Skin-on-a-Chip Translational Medicine Center, Joint Laboratory of Plastic Surgery Hospital, Chinese Academy of Medical Sciences and Southeast University, Beijing 100144, China; State Key Laboratory of Digital Medical Engineering, Institute of Microphysiological Systems, School of Biological Science and Medical Engineering, Southeast University, Nanjing 210096, China; Plastic Surgery Hospital, Chinese Academy of Medical Sciences and Peking Union Medical College, Beijing 100144, China; Skin-on-a-Chip Translational Medicine Center, Joint Laboratory of Plastic Surgery Hospital, Chinese Academy of Medical Sciences and Southeast University, Beijing 100144, China

**Keywords:** pigmentary disorders, *in vitro* skin models, 3D skin equivalents, skin-on-a-chip, tissue engineering

## Abstract

Cutaneous pigmentary disorders, including vitiligo, melasma, post-inflammatory hyperpigmentation (PIH) and solar/senile lentigines (SL), are prevalent, chronic and recurrent conditions driven by interactions among melanocytes, keratinocytes, fibroblasts, immune cells, the extracellular matrix (ECM) and environmental stimuli. Conventional animal models and two-dimensional cultures incompletely reproduce the cellular heterogeneity, tissue architecture and the dynamic microenvironment that regulates human pigmentation. To address these challenges, this review evaluates human-relevant *in vitro* models through a framework integrating biomaterial engineering, disease-specific modeling and translational evaluation, based on principles of tissue engineering, pathological reconstruction and predictive assessment. We compare *ex vivo* explants, co-cultures, three-dimensional skin equivalents, bioprinted constructs, organoids and skin-on-a-chip platforms in terms of cell sources, engineered matrices, structural fidelity, functional readouts, disease specificity, reproducibility and suitability for mechanistic studies and therapeutic evaluation. We further distinguish pigmentation-capable systems from models that recapitulate disease-specific pathological processes, including immune-mediated melanocyte loss in vitiligo, chronic dermal-epidermal signaling alterations in melasma, inflammation-associated pigmentation in PIH and senescence-related microenvironmental remodeling in SL. Current bottlenecks, including vascular and immune integration, long-term stability, quantitative monitoring, reproducibility, standardization and regulatory acceptance within the framework of New Approach Methodologies, are discussed.

## Graphical abstract

**Graphical Abstract:**
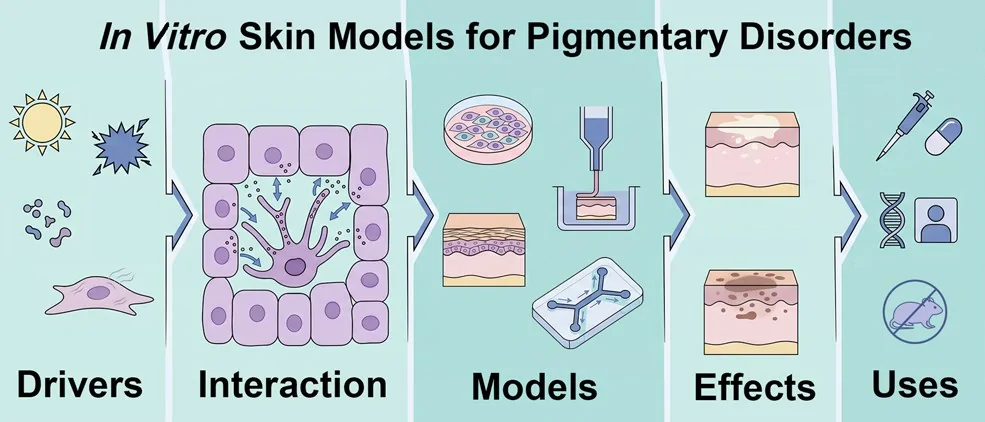

## Introduction

Cutaneous pigmentary disorders, including vitiligo, melasma, post-inflammatory hyperpigmentation (PIH) and solar/senile lentigines (SL), are characterized by high prevalence and recurrence rates worldwide. These conditions not only markedly impair physical appearance and psychosocial well-being but also impose a substantial long-term economic and public health burden [1]. Clinically, these disorders are typically chronic, relapsing and highly heterogeneous in phenotype. Their onset and progression involve complex interactions among multiple factors, including genetic susceptibility, endocrine and sex hormone fluctuations, chronic inflammation and immune dysregulation, oxidative stress and ultraviolet (UV) exposure. Meanwhile, intricate crosstalk among melanocytes, keratinocytes, fibroblasts and diverse immune cell populations further complicates the regulation of pigment homeostasis. Despite the availability of existing therapeutic strategies—such as phototherapy, topical agents and laser-based combination treatments—overall efficacy remains limited, and recurrence rates are high. Moreover, some interventions may even induce new pigmentary lesions, underscoring the urgent need for a systematic elucidation of the underlying mechanisms of pigmentary diseases from basic research to translational applications [2, 3]. However, the multifactorial and microenvironment-dependent nature of pigmentary disorders presents substantial challenges for conventional experimental systems, highlighting the need for human-relevant models capable of reproducing disease-associated cellular interactions and tissue-level regulation.

Against the backdrop of rapid advances in cosmetic and dermatological drug development, toxicological safety assessment and regulatory initiatives promoting New Approach Methodologies (NAMs), human cell-based *in vitro* skin models have increasingly been recognized as important alternatives to animal experimentation. Compared with conventional rodent models, advanced *in vitro* skin systems more closely recapitulate key physiological features of human skin, including keratinocyte differentiation, melanosome biogenesis and transfer, epidermal barrier function and inter-individual and ethnicity-related pigmentation differences. Consequently, such models have emerged as central platforms for investigating skin pigment homeostasis and the pathogenesis of pigmentary disorders [3, 4]. With continuous technological progress, *in vitro* skin models have progressed from early two-dimensional (2D) monolayer cultures of keratinocytes or melanocytes to multicellular reconstructed epidermis, full-thickness skin equivalents, skin organoids and microfluidic skin-on-a-chip systems. These multiscale platforms provide versatile and increasingly controllable experimental platforms for modeling skin aging, inflammatory responses and pigmentary dysregulation. However, increasing model complexity does not necessarily translate into improved biological relevance, and the selection of an appropriate platform should depend on the specific biological question, pathological mechanism and intended translational application.

From an application perspective, melanin-related *in vitro* models have demonstrated broad potential in elucidating disease mechanisms, drug and cosmetic screening and personalized pigmentary research. First, in mechanistic studies, three-dimensional (3D) pigmented skin models integrating melanocytes, keratinocytes, fibroblasts, and, in some cases, immune cells have been widely employed to systematically investigate the effects of UV radiation, oxidative stress and inflammatory mediators on melanogenesis and melanosome transfer. These models provide experimental platforms that more closely recapitulate the *in vivo* physiological environment, thereby facilitating the investigation of key pathogenic processes underlying vitiligo, melasma, PIH and SL. Second, in efficacy and safety evaluation, reconstructed pigmented epidermis or full-thickness skin models have been widely adopted by research institutions and testing centers as standardized tools for screening whitening, depigmenting and photoprotective ingredients. Endpoints such as macroscopic skin color changes, histological staining and quantitative assessment of melanin content enable the evaluation of melanogenesis-inhibitory or -stimulatory effects, while concurrent assessment of barrier function and irritation improves the robustness and translational relevance of test outcomes [5–7]. Third, considering that clinical drugs and topical formulations may elicit differential responses across populations with distinct pigmentation backgrounds, recent studies have advocated the use of *in vitro* skin models with varying baseline pigmentation levels to systematically evaluate the influence of skin pigmentation on percutaneous absorption and pharmacokinetics/pharmacodynamics (PK/PD). This strategy may ultimately enhance the equity and extrapolative validity of drug development and toxicological assessment [8]. Nevertheless, many existing models remain primarily optimized for reproducing pigmentation phenotypes rather than disease-specific pathological processes or clinically predictive responses. Therefore, a systematic evaluation of model capability, disease relevance and translational applicability is required to guide the development of next-generation platforms.

This review advances the current literature by shifting the focus from a platform-based classification of model formats to a fit-for-purpose framework for pigmentary disease modeling. Specifically, we integrate three dimensions that are often discussed separately: biomaterial design and tissue-engineering principles that shape skin-model fidelity, the disease-specific pathological features that must be reconstructed for different pigmentary disorders, and validation strategies required for drug development, cosmetic testing and NAMs. Figure 1 provides an overview of the conceptual framework and application landscape discussed in this review.

**Figure 1:**
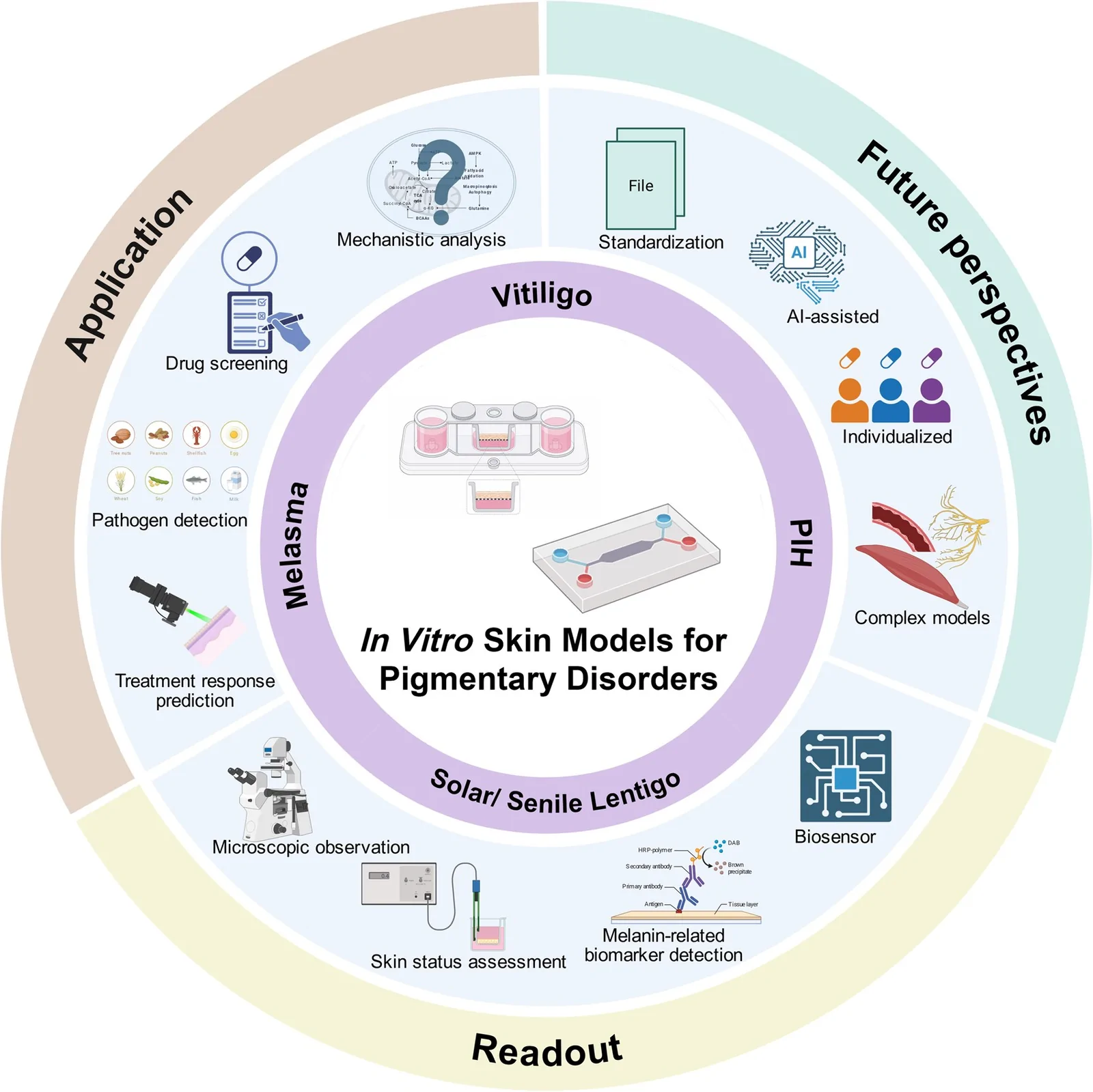
Construction and applications of *in vitro* models for cutaneous pigmentary disorders.

## Overview of the mechanisms of cutaneous pigmentation

### Melanin biosynthesis and metabolism

Melanin is a class of complex biopolymers derived from the amino acid tyrosine and is typically brown to black in color. By absorbing and scattering light across a broad range of wavelengths, melanin determines skin, eye and hair color in humans and many other animals [9]. Melanin is synthesized within melanocytes and stored in specialized lysosome-related organelles known as melanosomes, where it exerts a critical photoprotective function against UV-induced damage [10].

Melanin synthesis is a tightly regulated, multistep biochemical process that involves the coordinated action of key enzymes, transcription factors and signaling pathways. The process is initiated by the oxidation of L-tyrosine to dopaquinone catalyzed by tyrosinase (TYR), a reaction widely regarded as the rate-limiting step of melanogenesis. Subsequently, dopaquinone is further processed through a series of enzymatic reactions involving tyrosinase-related protein 1 (TYRP1) and dopachrome tautomerase (DCT, also known as TYRP2), ultimately giving rise to distinct types of melanin. These two enzymes collectively contribute to the synthesis of eumelanin and pheomelanin and regulate their relative proportions and overall production [11].

At the molecular level, the microphthalmia-associated transcription factor (MITF), first identified by Hodgkinson *et al.* [12], is widely recognized as a master regulator of melanocyte differentiation and melanogenesis. α-MSH/MC1R signaling elevates intracellular cAMP and activates PKA-CREB signaling, which promotes MITF expression and MITF-dependent transcription of TYR, TYRP1 and DCT/TRP2 [13–15]. MITF is also modulated by MAPK/ERK, WNT/β-catenin and PKC-related pathways, but these pathways should be interpreted as context-dependent regulatory modules rather than a single linear melanogenic cascade [11, 15].

Within the epidermis, melanocytes are primarily located in the basal layer. Their dendritic processes extend into suprabasal layers and establish stable functional complexes with approximately 30–40 surrounding keratinocytes, collectively referred to as the epidermal melanin unit [16]. How melanin is transferred from melanocytes to keratinocytes remains incompletely resolved, and multiple hypotheses have been proposed based on different experimental models [10]. Using transmission electron microscopy to examine human 3D *in vitro* skin models, Hall *et al.* [5] proposed that transfer of the melanosome core to keratinocytes via an exocytosis–phagocytosis pathway represents a predominant mechanism of melanin transfer in human epidermis, referred to as the exocytosis/phagocytosis model of melanin transfer.

Keratinocyte phagocytic uptake of melanin is closely regulated by protease-activated receptor-2 (PAR-2), a G protein-coupled receptor that is highly homologous to PAR-1, PAR-3 and PAR-4. Activation of PAR-2 by trypsin or specific peptide agonists markedly enhances melanin uptake by keratinocytes [17], partly through signaling pathways involving Rho family GTPases and adenylate cyclase [18]. UVB radiation has been shown to enhance pigmentation at least in part through activation of PAR-2 signaling; conversely, inhibition of PAR-2 with serine protease inhibitors results in skin lightening and significantly attenuates UVB-induced pigmentation responses [17]. In addition to PAR-2, keratinocyte growth factor [19] and Toll-like receptor 3 (TLR3) [20] can also enhance keratinocyte phagocytic activity via activation of Rho family GTPases.

Melanin particles internalized by keratinocytes accumulate in the perinuclear region, forming supranuclear structures known as microparasols that shield nuclear DNA from UV-induced damage [21]. Recent work suggests that transferred melanin can persist within low-degradative keratinocyte compartments for prolonged periods [22, 23]. Overall pigment clearance is therefore governed by both epidermal turnover and intracellular processing, including selective autophagy-related pathways under specific conditions [24, 25].

### Influence of the melanocyte microenvironment on melanogenesis

Keratinocytes exert sustained and multifaceted regulatory effects on melanocyte physiology through the secretion of paracrine factors, metabolites and cell–cell contact-dependent mechanisms. These regulatory interactions extend across the melanogenic process, including melanin synthesis, melanosome transfer, melanocyte survival and responses to UV-induced damage [26]. Keratinocyte-derived α-melanocyte-stimulating hormone (α-MSH) and adrenocorticotropic hormone (ACTH) activate the MC1R–cAMP–MITF signaling axis in melanocytes, thereby upregulating the expression of tyrosinase and tyrosinase-related protein 1 (TRP1), promoting eumelanin synthesis, enhancing dendrite formation and facilitating melanosome transfer to keratinocytes [27]. In parallel, keratinocyte-secreted endothelin-1 (ET-1) stimulates melanocyte proliferation, migration and melanogenesis via activation of the endothelin B receptor (EDNRB) and acts synergistically with α-MSH to upregulate MC1R expression [28]. Upon UV exposure, keratinocytes display increased production of prostaglandins such as PGE2 and PGF2α, as well as nitric oxide (NO). These mediators promote eumelanin synthesis by enhancing melanocyte dendrite extension, activating tyrosinase activity and stimulating cGMP-dependent signaling pathways, respectively [29, 30]. In contrast, keratinocyte-derived BMP-4, IL-1α, IL-6 and TNF-α exert inhibitory regulatory effects on melanogenesis by suppressing MITF or tyrosinase expression [31]. During photodamage responses, α-MSH, ET-1, PGE2 and NO also act cooperatively to enhance melanocyte DNA repair capacity. For example, α-MSH promotes activation of xeroderma pigmentosum group C (XPC) and XPA proteins and accelerates cyclobutane pyrimidine dimer (CPD) repair [32], while ET-1 reduces melanocyte apoptosis by enhancing nucleotide excision repair (NER) and activating Akt/Bcl-2 signaling [33]. In addition, by phagocytosing melanosomes and forming supranuclear melanin caps, keratinocytes themselves acquire additional UV-protective capacity.

Fibroblasts regulate melanocyte proliferation, differentiation and melanogenesis via the secretion of paracrine factors and the deposition of regulatory extracellular matrix (ECM) components. Early studies demonstrated that fibroblast-conditioned media and ECM constituents promote melanocyte proliferation and enhance tyrosinase activity [34]. In acral skin, fibroblasts exhibit elevated expression of dickkopf-1 (DKK1), which suppresses melanogenesis by inhibiting Wnt/β-catenin signaling and downregulating MITF, DCT and TYR expression [35]. Conversely, Wnt modulators such as secreted frizzled-related protein 2 (sFRP2) activate the Wnt/β-catenin–MITF pathway to stimulate melanogenesis and are upregulated following UV exposure [36]. Fibroblasts derived from darkly pigmented skin express elevated levels of neuregulin-1 (NRG-1), which promotes melanocyte proliferation and melanogenic responses through ErbB3/ErbB4–PI3K/Akt signaling [37]. Keratinocyte growth factor (KGF) secreted by fibroblasts enhances tyrosinase expression, increases keratinocyte phagocytic uptake of melanosomes, and acts synergistically with basic fibroblast growth factor (bFGF), ET-1 and cAMP signaling to amplify melanogenesis [38]. In contrast, the pleiotropic factor pleiotrophin (PTN) suppresses melanogenesis by promoting ERK1/2-mediated MITF degradation; its downregulation under UV conditions may contribute to tanning responses [39]. Moreover, fibroblast-derived CCN1 activates ERK/p38 MAPK signaling via integrin α6β1 to stimulate melanogenesis and is upregulated following UV exposure [40]. Collectively, fibroblasts modulate melanocyte function through a dynamic balance between pro-melanogenic and anti-melanogenic factors, thereby contributing to mechanisms underlying acral hypopigmentation, UV-induced tanning, skin aging and multiple pigmentary disorders. Additional regulatory mediators jointly secreted by fibroblasts and keratinocytes include hepatocyte growth factor (HGF), stem cell factor (SCF) and semaphorin 7A (SEMA7A) [26].

Beyond keratinocytes and fibroblasts, multiple skin-resident cell types and tissues participate in the fine-tuning of melanogenesis. The immune system plays a central role: IFN-γ, TNF-α, IL-17 and IL-22 secreted by CD4⁺ and CD8⁺ T cells suppress MITF and melanogenic gene expression and induce melanocyte apoptosis, thereby constituting key pathogenic mechanisms in vitiligo and PIH [41, 42]. Under stress conditions, Langerhans cells and dendritic cells may be activated by signals such as heat shock protein 70 (HSP70), thereby amplifying local anti-melanocyte immune responses [38]. Macrophages phagocytose melanin to form melanophages in inflamed or photoaged skin, a histopathological hallmark of PIH and SL. Importantly, macrophage polarization exerts differential effects on pigmentation: M1 macrophages inhibit melanogenesis via IL-1β, IL-6 and TNF-α, whereas M2 macrophages promote melanin synthesis during tissue repair by secreting vascular endothelial growth factor (VEGF), contributing to hyperpigmentation in scarred areas [43]. Vascular endothelial cells promote melanocyte proliferation and melanogenesis by secreting endothelin-1 (ET-1) and VEGF, which are robustly upregulated during the early stages of UV-induced pigmentation [44]. The nervous system is also actively involved in pigmentation regulation: peripheral sensory nerves release neuropeptides such as substance P and calcitonin gene-related peptide (CGRP), which modulate melanocyte survival, migration and MITF activity. Moreover, the skin itself functions as a peripheral neuroendocrine organ capable of locally synthesizing α-MSH, ACTH and other hormones that enhance melanogenesis via MC1R–cAMP signaling [45]. Adipocytes and dermal white adipose tissue influence melanocyte metabolism and migration through leptin, fibroblast growth factor 21 (FGF21) and stromal cell-derived factor 1 (SDF-1)/CXCR4 signaling, thereby contributing to age-associated pigmentary alterations [46]. The cutaneous microbiome may further influence pigmentation phenotypes by inducing oxidative stress and activating the aryl hydrocarbon receptor (AhR) pathway through microbial metabolites [47]. In addition, systemic endocrine hormones—including estrogens, thyroid hormones and insulin-like growth factor 1 (IGF-1)—modulate melanosome biogenesis and transfer via multiple signaling pathways [48]. When the basement membrane is structurally compromised, melanocytes may descend into the dermis, allowing melanin to be engulfed by dermal macrophages and leading to deeper and more persistent dermal pigmentation. This process represents an important pathological basis for PIH and certain forms of SL [49, 50]. Figure 2 summarizes the biological processes and microenvironmental signals most commonly reconstructed in pigmented *in vitro* skin models.

**Figure 2:**
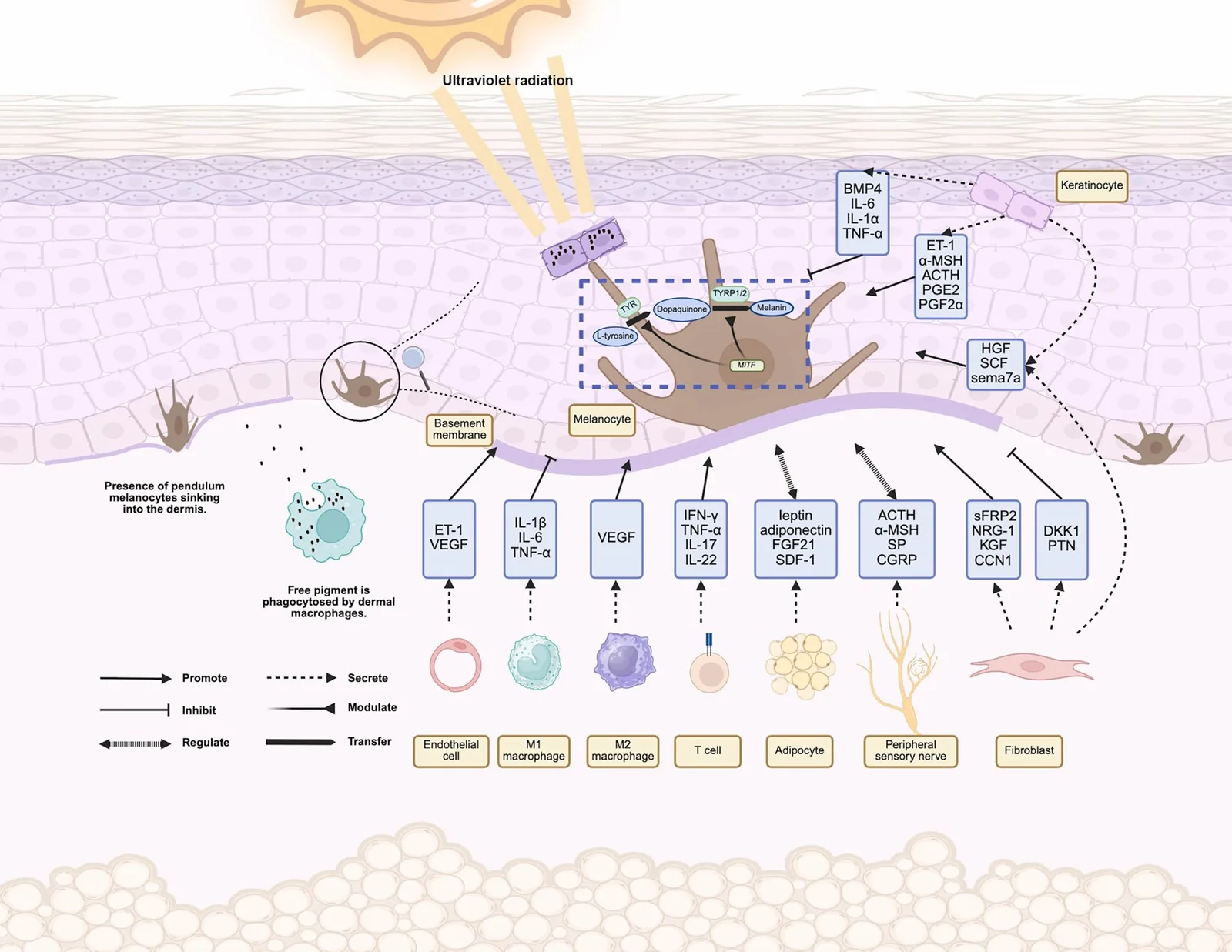
Melanin synthesis, distribution and regulatory mechanisms within the cutaneous microenvironment. Shown are melanosome biogenesis and maturation within melanocytes, their intercellular transfer to keratinocytes and the regulation of melanin homeostasis by multiple skin cell types and microenvironmental signals. Normal and aberrant distributions of melanocytes in the skin are illustrated. These key processes constitute the core biological events most commonly reconstructed and evaluated in *in vitro* pigmented-skin models.

## Pigmented *in vitro* skin models: classification, engineering design and evaluation metrics

In pigmentary disease modeling, biomaterials should be considered active regulators rather than passive cell carriers. Matrix stiffness, ligand presentation, degradation rate, porosity, oxygen diffusion, contraction resistance and optical compatibility can influence the establishment of a physiologically relevant pigmentary microenvironment by regulating melanocyte adhesion, dendricity, melanosome transfer, keratinocyte differentiation, fibroblast-derived paracrine signaling and image-based pigmentation readouts. Therefore, the translational value of pigmented skin models depends not only on cellular composition but also on whether the engineered extracellular microenvironment reproduces relevant mechanical, biochemical and transport features of human skin.

For this reason, the following sections distinguish model formats according to biological fidelity, engineering controllability and translational purpose. Different platforms occupy distinct positions within this framework: *ex vivo* tissues provide high native complexity but limited standardization, 2D cultures support high-throughput mechanistic screening, reconstructed and bioprinted models balance architecture with controllability, organoids capture developmental self-organization and skin-on-a-chip systems add perfusion, sensing and compartmentalized exposure.

### *Ex vivo* skin models

*Ex vivo* skin models constitute an intermediate experimental platform between conventional 2D cell culture systems and *in vivo* experiments. These models typically utilize full-thickness human skin obtained from plastic or dermatologic surgical procedures and are maintained either under air–liquid interface (ALI) or fully submerged culture conditions for extended periods. By preserving the native epidermal–dermal architecture, ECM composition and resident immune cell populations, *ex vivo* skin models more closely recapitulate skin barrier function, intercellular interactions and appendage biology. Consequently, they offer distinct interpretative advantages for applications such as irritation testing, barrier function studies, transdermal penetration and drug response evaluation [51, 52]. *Ex vivo* skin culture was first reported by Siddiqui *et al.* [53] in 1974 and represents one of the earliest skin research approaches with high physiological relevance. In recent years, advances in serum-free culture systems, oxygenation control, small interfering RNA (siRNA)-mediated gene silencing and reinnervation techniques have progressively extended the viable culture lifespan of *ex vivo* skin and broadened its range of applications. Nevertheless, this model is associated with inherent limitations, including restricted availability of clinical skin samples, difficulty in achieving standardized large-scale production, gradual degeneration of skin appendages during long-term culture and limited feasibility for genetic manipulation. In addition, skin tissue inevitably undergoes trauma and exposure to a hyperoxic environment during the explantation process, potentially inducing reactive oxygen species generation and wound-repair responses; these factors should be carefully considered when interpreting experimental data [51].

### Two-dimensional culture systems

Monocultures of melanocytes represent one of the most basic and widely used *in vitro* model systems for investigating the regulation of melanogenesis. By maintaining melanocytes as a monolayer in culture dishes, these systems allow direct examination of intracellular melanosome biogenesis and maturation, as well as the regulatory networks governing MITF and its downstream tyrosinase-family genes. In addition, they are particularly well suited for assessing the direct effects of drugs, small molecules, UV radiation or oxidative stress on melanocyte function [54]. The earliest methods for melanocyte culture date back to the work of Eisinger and Marko [55] in 1982, who successfully isolated and expanded human melanocytes from epidermal single-cell suspensions *in vitro*, laying the foundation for modern human melanocyte culture systems. Subsequently, Le Poole *et al.* [56] established the first stable immortalized human epidermal melanocyte cell line through the introduction of HPV16 E6/E7 genes, thereby providing a valuable resource for long-term and reproducible *in vitro* studies. Melanocytes can also be co-cultured with keratinocytes at defined ratios to partially reconstitute the intercellular interactions within the epidermal melanin unit. Such co-culture systems offer advantages, including operational simplicity, low cost and high experimental controllability, and have therefore become widely used platforms for studying melanogenesis, dendrite formation, melanosome transport, drug responses and photobiological effects [6, 54–56]. Notably, Gledhill *et al.* [57] successfully differentiated functional human melanocytes from induced pluripotent stem cells (hiPSCs) and co-cultured them with primary keratinocytes, thereby achieving effective melanosome transfer within a fully iPSC-derived human system. Overall, 2D melanocyte monoculture and melanocyte–keratinocyte co-culture systems remain important tools in pigmentation research due to their high throughput, strong controllability and suitability for mechanistic studies. A large body of research has relied on these 2D models to investigate the regulation of melanogenesis, screen candidate compounds, characterize UV responses and elucidate mechanisms underlying PIH.

### Three-dimensional pigmented skin equivalents

Three-dimensional pigmented skin equivalents are typically constructed by seeding melanocytes and keratinocytes at defined ratios (commonly 1:10–1:20) onto the surface of biomimetic skin-like matrices, such as fibroblast-containing collagen gels or de-epidermized dermis. Under ALI culture conditions, keratinocytes undergo stepwise differentiation to form a stratified epidermis comprising the basal, spinous, granular and cornified layers. Within this architecture, melanocytes are primarily localized to the basal layer and form functional epidermal melanin units with the overlying keratinocytes [58]. Compared with 2D culture systems, these models more closely recapitulate the *in vivo* skin state in terms of keratinocyte differentiation, melanosome biogenesis and transfer, barrier function and integrated responses to UV radiation and exogenous stimuli. As a result, 3D pigmented skin equivalents have been extensively applied to studies of human skin pigment homeostasis, tanning responses and pigmentary disorder–associated mechanisms [4, 58]. Importantly, the choice of dermal matrix is not merely a structural consideration but a critical determinant of cellular organization, paracrine signaling and long-term pigmentation stability.

The earliest 3D pigmented skin equivalent was reported by Topol *et al.* [59] in 1986. In this pioneering work, fibroblasts were embedded within collagen matrices to generate dermal equivalents, onto which human melanocytes were seeded. Subsequent coverage with keratinocytes derived from neonatal skin enabled the formation of a multilayered epidermis, resulting in a fully human, pigmented skin equivalent *in vitro*. Building upon this framework, Gledhill *et al.* [57] reported the first full-thickness pigmented 3D skin equivalents generated exclusively from hiPSC-derived keratinocytes, fibroblasts and melanocytes. Using reconstructed human pigmented skin and epidermal models, Hall *et al.* [5] further systematically investigated melanosome transfer processes and proposed that transfer of the melanosome core (melanocore) via melanocyte exocytosis followed by keratinocyte phagocytosis—referred to as ‘melanocore transfer’—constitutes a predominant mechanism underlying epidermal pigmentation. This mechanism was validated by both electron microscopy and confocal imaging, demonstrating a high degree of concordance between 3D skin equivalents and human skin biopsy specimens. Under ALI conditions, these 3D pigmented skin equivalents can be maintained in a stable state for several weeks and, in some cases, up to approximately 50 days, while preserving epidermal stratification and pigment distribution. Such stability enables dynamic monitoring of tanning, depigmentation and pharmacological interventions, as well as longitudinal analysis of melanosome fate and melanogenic responses over time. More recent full-thickness models based on fibroblast-derived matrices further demonstrate that matrix origin, scaffold contraction resistance, barrier maturation and long-term culture stability can determine whether melanocytes remain correctly positioned in the basal layer and whether constitutive pigmentation remains interpretable over time [60]. These parameters influence basement membrane organization, dermal-epidermal mechanical coupling and molecular diffusion, thereby affecting the interpretation of pigmentation and inflammatory phenotypes.

### Three-dimensional bioprinted skin models incorporating melanocytes

Three-dimensional bioprinting offers an advanced bioengineering strategy for constructing skin models with both hierarchical architecture and functional heterogeneity. As early as 2003, Mironov *et al.* [61] proposed the concept of ‘organ printing’, systematically demonstrating the feasibility of computer-assisted, layer-by-layer fabrication of living tissues and organs using jet-based printing approaches, thereby establishing a theoretical foundation for the subsequent development of 3D bioprinting technologies. Building on this concept, Lee *et al.* [62] demonstrated the feasibility of fabricating human skin–like constructs by multilayer deposition of human dermal fibroblasts and keratinocytes onto biodegradable scaffolds using 3D printing techniques, thereby providing important technical groundwork for the practical implementation of bioprinted skin models. On this basis, Min *et al.* [63] further introduced melanocytes into 3D bioprinted skin systems and successfully generated biomimetic full-thickness skin equivalents containing melanocytes. In their approach, extrusion-based bioprinting was used to deposit fibroblast-laden collagen hydrogels layer by layer, which were subsequently neutralized and crosslinked with sodium bicarbonate to form the dermal compartment. Melanocytes and keratinocytes were then sequentially printed onto the dermal layer and cultured under ALI conditions, allowing keratinocytes to undergo differentiation and cornification and to form a functional stratum corneum. Histological and pigmentation analyses revealed that these constructs not only exhibited well-defined epidermal–dermal stratification but also displayed discretely distributed pigmented spots at the epidermal–dermal junction, resembling freckle-like epidermal pigmentation that emerged spontaneously in the absence of UV irradiation or exogenous chemical stimulation. Kang *et al.* [64] subsequently developed a multimodal 3D bioprinting strategy integrating extrusion-based printing with microvalve control. Through algorithm-driven optimization of nozzle movement and droplet deposition, they achieved precise spatial coordination of melanocyte placement, enabling spatially programmable array-based deposition of melanocytes within skin constructs. This approach allowed simultaneous control over melanocyte spatial distribution and pigmentation density within a single model, resulting in either homogeneous or patterned pigment distribution. Moreover, the incorporation of functional bioinks endowed the constructs with anti-inflammatory, antibacterial and antioxidative properties, thereby conferring protective effects against inflammatory and oxidative stress conditions in Transwell co-culture systems. From a materials-design perspective, different bioink systems based on collagen, fibrin, gelatin/GelMA, hyaluronic acid, decellularized ECM and composite hydrogel provide distinct advantages in terms of printability, crosslinking chemistry, contraction control, cell-remodeling capacity, diffusion properties and compatibility with optical or histological pigmentation readouts. These parameters should be reported together with cell ratios and ALI culture conditions because scaffold mechanics and mass transport can confound pigmentation, barrier and inflammatory endpoints and limit cross-study reproducibility [65].

### Skin organoids incorporating melanocytes

Organoid technology was first introduced by Sato *et al.* [66] in 2009, who demonstrated that adult stem cells are capable of self-organizing into organoids with defined structural and functional features when cultured in 3D matrices, thereby establishing the conceptual and methodological foundation of modern organoid systems. Building on these principles, Lee *et al.* [67] successfully generated human skin organoids from hiPSCs by stepwise modulation of transforming growth factor-β (TGF-β) and fibroblast growth factor (FGF) signaling pathways. Through long-term culture over approximately 4–5 months, these 3D spheroidal aggregates underwent coordinated differentiation of cranial ectoderm and neural crest lineages, thereby self-organizing into complex skin organoids containing stratified keratinized epidermis, adipocyte-rich dermal-like mesenchyme, pigmented hair follicles and sebaceous glands, as well as sensory neurons and glial cells. This seminal work provided the first direct evidence that human pluripotent stem cells, when exposed to appropriate morphogenetic signaling environments, are capable of spontaneously reconstructing skin *in vitro* with functional appendages, including hair follicles and melanocytes. As such, skin organoids represent a physiologically relevant platform for modeling skin development and pigmentary diseases. Following the establishment of this robust differentiation framework, multiple subsequent studies have further refined and expanded the application of melanocyte-containing skin organoids, including refined characterization of the epidermal–dermal junction, disease modeling, infection platforms and regenerative transplantation strategies [68–70].

### Skin-on-a-chip models

Organ-on-a-chip technology is commonly traced to the landmark lung-on-a-chip reported by Huh *et al.* [71] in 2010, in which a microfluidic device incorporating controlled fluid shear stress and cyclic mechanical stretching recapitulated organ-level lung functions *in vitro*. Building on this paradigm, Atac *et al.* [72] developed one of the earliest skin-on-a-chip systems, termed skin and hair-on-a-chip, in which full-thickness skin equivalents and hair follicles were sequentially cultured within a dynamically perfused chip-based bioreactor. Under variable shear stress, this system prolonged culture while preserving hair follicle integrity and dermal-epidermal junction structure. Subsequently, Li *et al.* [7] developed a triple-well microfluidic epidermis-on-a-chip system that combined multichannel perfusion with ALI culture, supported epidermal stratification and barrier maturation and incorporated a melanin-mimicking module for depigmenting cosmetic-agent evaluation. More recently, Barros *et al.* [73] integrated 3D bioprinting with microfluidic engineering to construct a vascularized human skin/skin cancer-on-a-chip model for localized microneedle-based drug testing.

From an engineering perspective, skin-on-a-chip systems provide three key advantages over static Transwell models: controlled perfusion, compartmentalized biochemical gradients and real-time or repeated monitoring. Flow rate, shear stress, membrane composition, channel geometry, oxygenation, sampling frequency, compound absorption into device materials and ALI stability should therefore be treated as experimental variables, particularly for topical drugs, cosmetic ingredients, nanoparticles, phototoxicity studies and inflammatory challenges [74, 75]. These parameters are particularly important for pigmentary disorders because melanogenesis, melanosome transfer, inflammatory signaling and pigment clearance are highly dependent on spatial and temporal microenvironmental regulation. For pigmentary disorders, chip designs should ideally separate epidermal, dermal, endothelial/vascular, immune and reservoir compartments while preserving ALI exposure, enabling disease-relevant modeling of UV stimulation, cytokine gradients, melanocyte loss, barrier disruption, dermal pigment retention and drug penetration.

Key unresolved barriers include the construction of perfusable microvasculature, controlled incorporation of immune cells without nonspecific activation, prevention of tissue contraction or delamination during long-term culture, reduction of PDMS-related absorption for hydrophobic compounds, harmonization of transepithelial electrical resistance (TEER)/permeability readouts across devices and standardization of chip geometry and acceptance criteria. These limitations explain why skin-on-a-chip platforms are promising next-generation models but have not yet replaced simpler models for routine pigmentary screening. Future development should therefore focus not on replacing existing platforms, but on establishing complementary model hierarchies in which each system is selected according to the specific biological question and translational objective. Figure 3 presents the developmental trajectory of pigmented *in vitro* skin models from simple cultures and *ex vivo* systems to reconstructed tissues, organoids and skin-on-a-chip/microphysiological systems (MPS) platforms.

**Figure 3:**
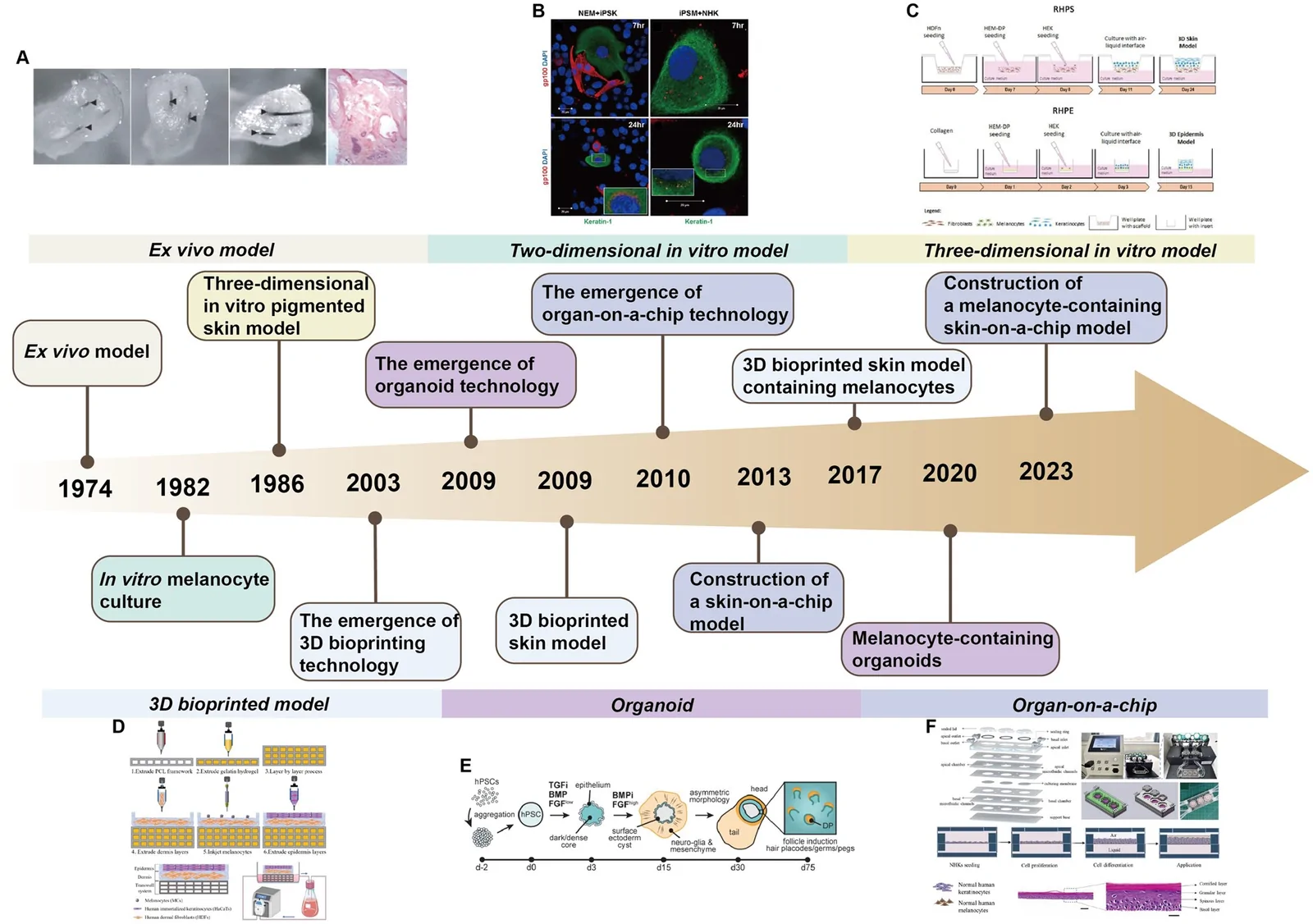
Developmental trajectory of pigmented *in vitro* skin models. (**A**) Long-term *ex vivo* skin culture [52]. (**B**) Keratinocyte-melanocyte co-culture [57]. (**C**) Reconstructed pigmented epidermis and full-thickness skin equivalents [5]. (**D**) Bioprinted melanocyte-containing skin constructs [64]. (**E**) Skin organoids containing appendage and pigment-lineage components [67]. (**F**) Epidermal or pigment-related microphysiological systems cultured on chip-based platforms [7].

### Cell sources and evaluation metrics

In the construction of melanin-containing *in vitro* skin models, the primary cell sources include commercially available human melanocyte and keratinocyte cell lines [5], primary melanocytes and other skin cell types isolated from skin biopsies or surgically discarded tissue [76], as well as melanocytes and epidermal cells differentiated from human induced pluripotent stem cells (hiPSCs) or embryonic stem cells (ESCs) in more recent studies [77]. Each cell source is associated with distinct advantages and limitations in terms of availability, phenotypic stability, pigmentation fidelity, genetic manipulability and translational relevance, which collectively influence model selection, experimental design and translational applicability, as summarized in Table 1 [78–81]. At the structural level, two main strategies have been reported: the direct use of commercially available pigmented reconstructed epidermis or full-thickness skin models with subsequent induction of disease-relevant or stimulatory conditions and secondary seeding of melanocytes onto non-pigmented commercial skin equivalents to generate pigmented models *de novo* [82, 83]. Functional evaluation of melanin-related skin models generally encompasses multiple readouts, including total melanin content, tyrosinase activity, expression of key genes and proteins along the MITF–TYR–TYRP1/2 axis, as well as phenotypic assessment of melanosome biogenesis and intercellular transfer [25, 57]. In addition, comprehensive evaluation of overall tissue architecture and maturation requires the integration of histological and immunohistochemical analyses with barrier function assessments, such as TEER and transdermal permeability measurements [84–86]. Together, Tables 1–3 provide complementary perspectives by summarizing cell sources, evaluation criteria and platform-level characteristics, respectively. To further support fit-for-purpose model selection, Table 3 explicitly compares major model formats not only by biological relevance and best-fit application, but also by throughput, cost, culture duration, reproducibility, scalability and genetic tractability.

**Table 1:** Comparison of different cell sources used in pigmented *in vitro* skin models.

Comparison dimension	Commercial melanocyte cell lines	Primary melanocytes	iPSC-derived melanocytes
Biological functionality	Preserved basic melanogenic function; partial phenotypic drift	Highest physiological relevance; intact function; functional decline in long-term culture; stimulation may be required	Comparable to primary melanocytes; melanin synthesis/transfer
Consistency/stability	High consistency; low batch variation	Donor variability; batch fluctuation	Line-dependent consistency; quality-controlled stability
Ethical/regulatory aspects	Minimal ethical concerns	Donor consent; ethics approval required	Low ethical concern; reprogramming-based
Availability/scalability	Readily available; unlimited expansion	Limited supply; restricted expansion	Renewable source; scalable production
Genetic manipulability	High	Moderate to low	High
Technical complexity	Low	Moderate	High
Cost	Low	Moderate	High
Translational relevance	Moderate	High	High; personalized modeling
Main strengths	Standardized; robust; convenient	High physiological relevance	Expandable; patient-specific; engineerable
Main limitations	Reduced *in vivo* fidelity	Limited access; donor variation	Complex workflow; long differentiation

iPSC, induced pluripotent stem cell.

**Table 2:** Evaluation metrics for pigmented *in vitro* skin models.

	Metric	Significance	Measurement method	Reference(s)
Melanin-related evaluation metrics	Melanin content	Total melanin production level	Spectrophotometric quantification after melanin extraction (NaOH digestion with OD measurement); image-based quantification following Fontana–Masson staining	[25, 85]
	Tyrosinase activity	Enzymatic activity of tyrosinase in melanogenesis	L-DOPA oxidation assay for enzyme activity (colorimetric quantification of reaction products)	[85]
	Melanocyte dendricity	Dendrite extension capacity and melanosome delivery potential	Microscopic quantification of dendrite number/length (fluorescence microscopy or Fontana–Masson staining)	[85]
	Melanosome transfer and distribution	Efficiency and pattern of melanosome transfer to keratinocytes	Histological observation by Fontana–Masson staining; TEM; flow cytometry	[5, 6]
	Pigmentation-related genes/proteins	Molecular regulation of melanogenesis and melanosome transport	Measurement of mRNA or protein levels of MITF, TYR, TYRP1, α-MSH, etc. (qPCR, Western blotting, immunofluorescence)	[25]
	Signaling pathway activity	Activation status of melanogenesis-related signaling pathways	Determination of cAMP levels; p-CREB/CREB ratios (pathway-specific analyses such as Western blotting or ELISA)	[85]
Skin model structure and status metrics	Tissue architecture	Integrity of epidermal–dermal structure and stratification	H&E paraffin sections; Fontana–Masson staining to assess epidermal layer organization	[5]
	Stratification and differentiation markers	Epidermal differentiation status and layer-specific marker expression	IHC/IF detection of K14, K10, filaggrin, etc., to localize epidermal stratification	[5]
	Barrier function	Epidermal barrier integrity and permeability control	TEER measurements; transdermal diffusion assays; TEWL measurements; analysis of stratum corneum lipids and tight junction proteins	[84]
	Cell viability	Overall cell survival and metabolic activity	Viability assays such as MTT colorimetric assays (dye conversion absorbance); Live/Dead fluorescence staining	[85]
	Inflammatory markers	Inflammatory response and immune activation status	Measurement of cytokines such as IL-1α/IL-8 (ELISA); expression of inflammation-related genes (qPCR)	[86]
	Colorimetric parameters	Quantitative assessment of skin color intensity and uniformity	Colorimeter-based measurement of L*a*b* values to calculate the ITA; or image-based skin color analysis	[8]
Advanced validation metrics	AI analysis	Spatial melanin/tissue-quality quantification	Computer-vision analysis of pigmented-skin images	[87]
	Live imaging	Temporal pigment dynamics and drug response	Repeated standardized bright-field/fluorescence imaging	[87]
	On-chip sensors	Real-time barrier, metabolic and inflammatory status	TEER, impedance, oxygen, pH and cytokine readouts	[88]
	QC criteria	Analytical, biological and context-of-use validity	Predefined viability, barrier, pigmentation and benchmark thresholds	[88, 89]

OD, optical density; NaOH, sodium hydroxide; L-DOPA, L-3,4-dihydroxyphenylalanine; TEM, transmission electron microscopy; MITF, microphthalmia-associated transcription factor; TYR, tyrosinase; TRP1, tyrosinase-related protein 1; α-MSH, alpha-melanocyte-stimulating hormone; qPCR, quantitative polymerase chain reaction; cAMP, cyclic adenosine monophosphate; CREB, cAMP response element-binding protein; ELISA, enzyme-linked immunosorbent assay; H&E, hematoxylin and eosin; IHC, immunohistochemistry; IF, immunofluorescence; TEER, transepithelial electrical resistance; TEWL, transepidermal water loss; ITA, individual typology angle.

**Table 3:** Engineering and translational comparison of *in vitro* pigmented skin model platforms.

Model platform	Physiological/biological relevance	Engineering controllability, throughput, cost and culture duration	Reproducibility, scalability and genetic tractability	Best-fit applications	Key limitations/translational status	Reference(s)
Two-dimensional melanocyte or keratinocyte-melanocyte culture	Low to moderate; lacks 3D architecture and barrier	High control; high throughput, low cost and short duration	High reproducibility and scalability; high genetic tractability	Mechanistic screening; direct melanocyte responses	Weak tissue context; limited transfer and barrier fidelity; useful as early screen	[6, 54–56]
*Ex vivo* skin explant	High native architecture, ECM, appendages and resident cells	Low throughput; donor/sampling constraints; short viable culture window	Low to moderate reproducibility; poor scalability; limited genetic tractability	Validation against native tissue; lesion-specific responses	Restricted availability and short culture window; strong comparator but hard to standardize	[51–53]
Reconstructed pigmented epidermis/full-thickness skin	Moderate to high; stratification, basal melanocytes, barrier readouts	Moderate controllability, throughput, cost and days-to-weeks culture duration	Moderate to high reproducibility in standardized systems; moderate scalability and genetic tractability	Pigmentation biology, UV/drug testing, tissue-level depigmentation	Limited vasculature, nerves, immune cells and chronic remodeling; mature for standardized tests	[5, 57, 59, 60]
3D bioprinted pigmented skin	Moderate to high; spatial patterning and matrix architecture tunable	High spatial control; moderate throughput, high setup cost and maturation time	Reproducibility depends on bioink, printing parameters and maturation protocol; moderate scalability; moderate to high genetic tractability	Patterned pigmentation, material testing, scalable tissue fabrication	Bioink contraction, maturation time, diffusion limits and printing stress; process reporting needed	[61–65]
Skin organoid	High developmental complexity; appendage and neural components possible	Self-organizing; low throughput, high cost and long duration	Variable reproducibility; limited scalability; potentially high genetic tractability through iPSC/gene-editing workflows	Developmental pigmentation, genetic disease modeling, personalization	Variable maturity and incomplete vascular/immune control; powerful for biology but less screening-ready	[66–70]
Skin-on-a-chip/MPS	Potentially high if perfusion, ALI, barrier, immune/vascular modules align	High control of flow, gradients and sensors; low to moderate throughput and variable duration	Reproducibility limited by device/protocol heterogeneity; low to moderate scalability; genetic tractability depends on incorporated cells	Dynamic exposure, barrier monitoring, PK/PD-linked testing	Vascular/immune integration, long-term stability, device absorption and standardization remain limiting	[7, 71–75, 84, 87, 88, 90]

2D, two-dimensional; 3D, three-dimensional; ECM, extracellular matrix; UV, ultraviolet; MPS, microphysiological systems; ALI, air-liquid interface; PK/PD, pharmacokinetic/pharmacodynamic; iPSC, induced pluripotent stem cell.

## Molecular mechanisms and disease-specific *in vitro* models of pigmentary disorders

Cutaneous pigmentary disorders arise from disruptions in skin pigment homeostasis and can be broadly classified into depigmenting disorders and hyperpigmentary disorders according to the direction of melanin alteration. Although both categories involve a common cellular repertoire—including melanocytes, keratinocytes, fibroblasts and immune cells—their pathogenic mechanisms, dominant microenvironmental drivers and corresponding requirements for *in vitro* disease modeling differ substantially [26, 42]. In this review, we focus on four representative pigmentary disorders—vitiligo, melasma, PIH and SL—as prototypical conditions that collectively represent the major pathological axes of pigment dysregulation, encompassing depigmentation versus hyperpigmentation, acute inflammatory versus chronic microenvironment-driven processes and immune-, senescence- and UV-associated pathways. A critical conceptual distinction is therefore needed between pigmentation-capable models and disease-specific models. A model that produces melanin, supports melanosome transfer or responds to depigmenting agents is suitable for studying general melanogenesis, but it does not automatically recapitulate disease pathology. Accordingly, disease-specific models should be evaluated according to whether they reconstruct the initiating pathological trigger, the key cellular interactions involved and the microenvironmental changes that sustain disease progression. Vitiligo models require melanocyte stress, detachment, apoptosis, antigen presentation or immune-mediated killing; melasma models require sustained epidermal-dermal paracrine and vascular/basement-membrane alterations; PIH models require inflammatory coupling and, for dermal PIH, barrier-junction disruption with macrophage pigment uptake; and SL models require photoaging- or senescence-associated dermal remodeling.

### Depigmenting disorders

Figure 4 summarizes representative *in vitro* models of depigmenting disorders.

**Figure 4:**
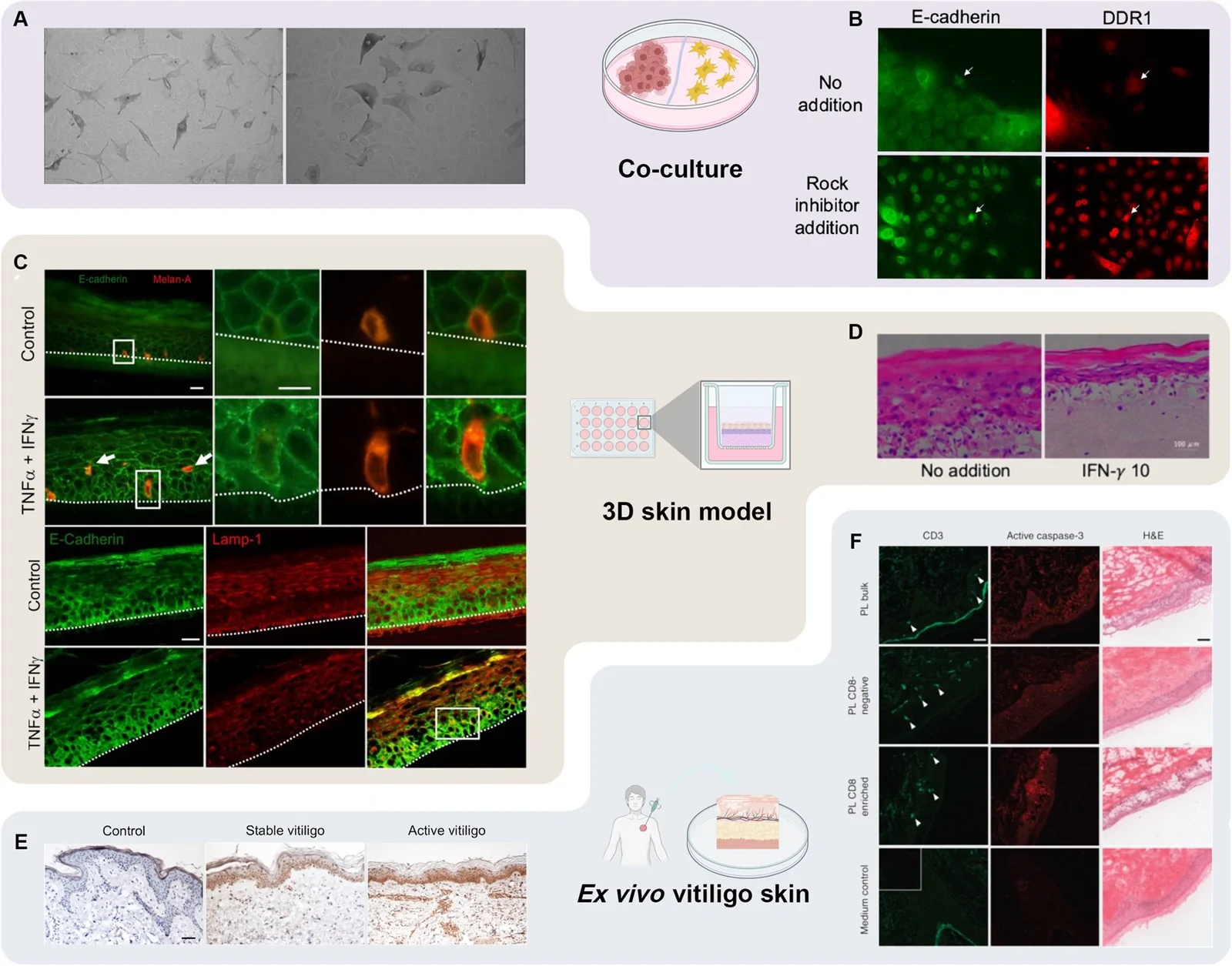
*In vitro* models of depigmenting disorders. (**A**) Healthy- and vitiligo-derived keratinocyte-melanocyte co-cultures [91]. (**B**) iPSC-derived melanocyte-keratinocyte systems [92]. (**C**) Cytokine-stimulated reconstructed pigmented epidermis showing adhesion-related changes [93]. (**D**) IFN-γ-triggered cell-death modeling in a 3D skin model [92]. (**E**) MMP-9 expression in control and vitiligo skin [93]. (**F**) Perilesional T-cell-mediated melanocyte killing in autologous skin explants [94].

#### Vitiligo

Depigmenting disorders are primarily characterized by a reduction or complete loss of epidermal melanin content. Their fundamental pathological basis lies not in a simple impairment of melanin synthesis, but rather in melanocyte instability, functional dysfunction or cell loss. Among these conditions, vitiligo represents the most extensively investigated prototype. Its pathogenesis involves the synergistic interplay of genetic susceptibility, oxidative stress and immune dysregulation, ultimately resulting in the selective destruction of melanocytes [41, 42]. Accordingly, *in vitro* modeling of depigmenting disorders has largely focused on recapitulating melanocyte vulnerability within inflammatory or immune-activated microenvironments. Key modeling priorities include the investigation of melanocyte–keratinocyte interactions, oxidative stress-induced melanocyte vulnerability, cytokine-mediated melanocyte detachment or apoptosis and cytotoxic effects driven by immune cells [91–96]. However, most existing vitiligo models remain limited in reproducing the coordinated interaction among oxidative stress, immune activation and tissue repair responses within a human skin microenvironment.

### Hyperpigmentary disorders

Hyperpigmentary disorders comprise a heterogeneous group of conditions characterized by excessive or persistent accumulation of melanin within the skin. Despite their diverse clinical manifestations, disorders such as melasma, PIH and SL share several common pathogenic features. These include sustained melanocyte activation, amplification of pro-melanogenic signaling, dysregulated melanosome transport and impaired pigment clearance, which together contribute to the maintenance of hyperpigmented lesions [97–100]. Figure 5 summarizes representative *in vitro* models of hyperpigmentary disorders.

**Figure 5:**
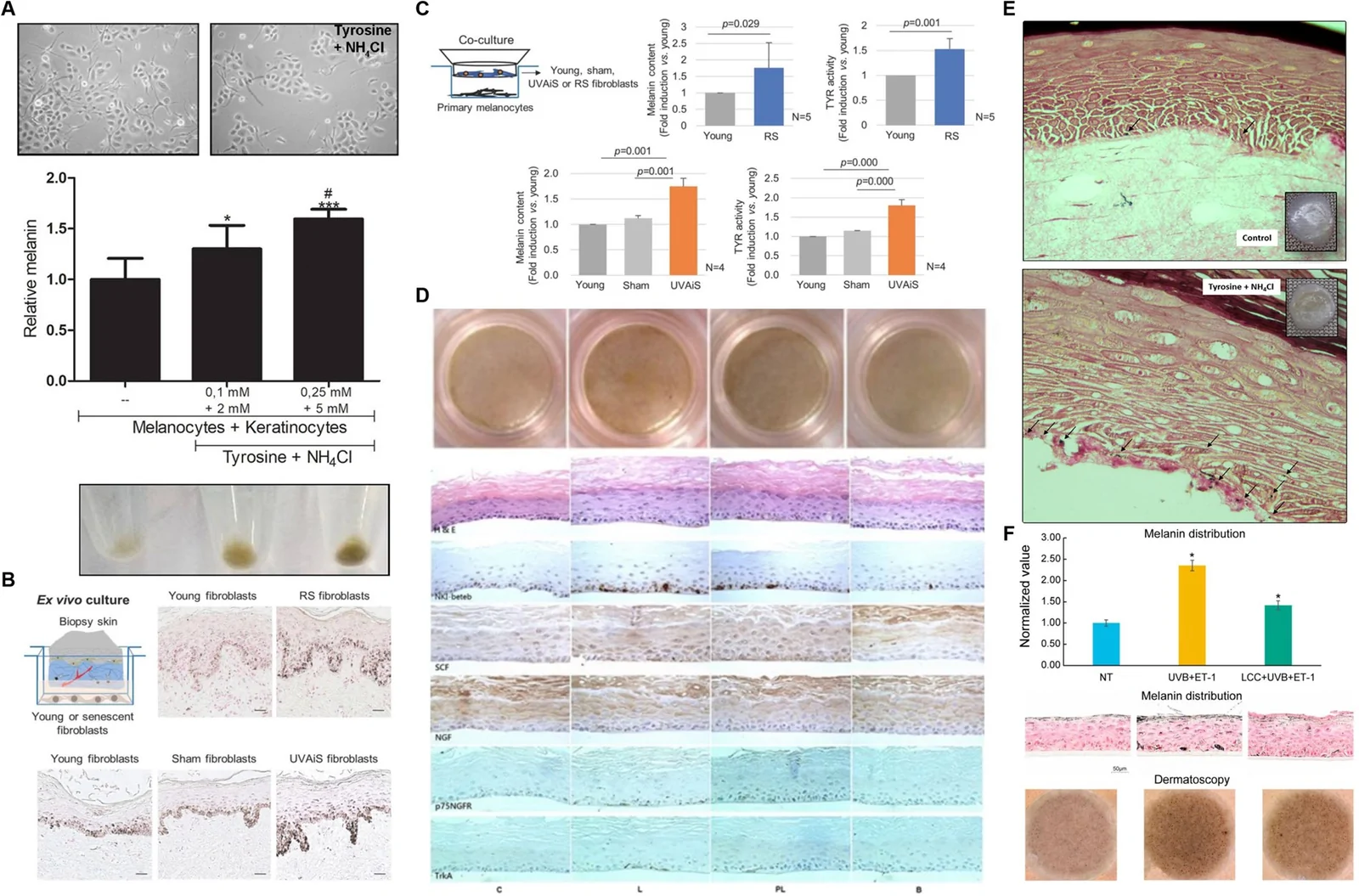
*In vitro* models of hyperpigmentary disorders. (**A**) Tyrosine/NH4Cl-induced melanogenesis in keratinocyte-melanocyte co-culture [101]. (**B**) Senescent-fibroblast models for SL-like pigmentation [102]. (**C**) *Ex vivo* validation showing that senescent fibroblasts enhance epidermal pigmentation [102]. (**D**) Melasma fibroblast-driven pigmentation in epidermal equivalents [82]. (**E**) Tyrosine/NH4Cl-induced pigmentation in 3D skin models [101]. (**F**) ET-1/UVB-induced lentigo-like pigmentation in a commercial 3D skin equivalent [83].

#### Melasma

Melasma is a multifactorial, chronic hyperpigmentation disorder, in which the core pathology extends beyond intrinsic melanocyte abnormalities to involve long-term dysregulation of epidermal–dermal microenvironmental crosstalk. Ultraviolet radiation, hormonal influences and genetic predisposition collectively drive dermal fibroblasts, keratinocytes and vascular-associated structures to continuously secrete pro-melanogenic paracrine factors, thereby maintaining melanocytes in a persistently activated state. Concurrently, alterations in basement membrane integrity and remodeling of dermal components facilitate the stable retention of pigment within lesional areas, providing a mechanistic basis for the high recurrence rate and therapeutic refractoriness of melasma [97]. Accordingly, *in vitro* models of melasma have predominantly emphasized the role of lesion-associated fibroblasts or *ex vivo* skin microenvironments in sustaining hyperpigmented states, rather than transient melanogenic responses induced by acute stimulation alone [82, 98]. This distinction is critical because short-term melanogenic activation may reproduce pigmentation changes but fails to capture the chronic and recurrent nature of melasma.

#### Post-inflammatory hyperpigmentation

PIH represents a common secondary pigmentary phenotype that develops following cutaneous inflammation or injury. Its defining feature is the aberrant coupling between inflammatory signaling and melanogenic pathways. Inflammatory mediators can directly or indirectly activate the MITF-dependent melanogenic program in melanocytes while simultaneously promoting melanosome transfer to keratinocytes. When inflammation is accompanied by disruption of the epidermal–dermal junction, melanin may descend into the dermis and be phagocytosed by macrophages, resulting in deeper and more slowly resolving pigmentation [99, 100]. Accordingly, *in vitro* modeling of PIH typically aims to recapitulate the combined effects of inflammatory stimulation and structural tissue damage, with particular emphasis on distinguishing the distinct formation pathways of epidermal-type and dermal-type hyperpigmentation and macrophage-mediated pigment retention [101, 103].

#### Solar/senile lentigo

SL is a chronic pigmentary lesion arising in photoaged skin, in which the defining feature is the persistent remodeling of pigment homeostasis by a senescence-associated microenvironment. Prolonged UV exposure induces senescence in both keratinocytes and dermal fibroblasts, accompanied by sustained release of pro-inflammatory and pro-melanogenic factors that maintain elevated local melanogenic activity. At the structural level, degeneration of the epidermal–dermal junction, remodeling of the dermal ECM and macrophage-mediated pigment retention collectively contribute to the long-term stability of SL [49, 102, 104–106]. Consequently, *in vitro* models of solar/senile lentigo have primarily focused on senescent cell–driven pigment dysregulation, rather than acute UV stimulation alone [83, 102, 107–109]. This highlights the importance of incorporating aging-associated cellular states and extracellular matrix remodeling when developing SL models. Representative pathological mechanisms and disease-specific *in vitro* models are summarized in Table 4.

**Table 4:** Major pathological mechanisms and representative *in vitro* models of cutaneous pigmentary disorders.

Disease	Major triggers	Key pathological mechanisms	Major involved cells/Structures	Representative studies	Year	Model type	Cells/Structures included in the model	Pathological features recapitulated/Validated in the model	Reference(s)
SL	Chronic UV exposure; cellular senescence	Chronic UV and senescence reshape fibroblast SASP, amplifying melanogenic signaling. Matrix remodeling, inflammation, basement membrane disruption and macrophage-driven pigment retention sustain hyperpigmentation.	Melanocytes; senescent fibroblasts; dermal macrophages; epidermal–dermal junction	Cai *et al.*	2025	3D	MelaKutis® (pigmented 3D living skin equivalent)	ET-1 (± UVB) induces reproducible hyperpigmented phenotypes and upregulates TYR and related pathways	[83]
				Meunier *et al.*	2023	*Ex vivo*	Normal human skin	H₂O₂/UV treatment establishes SL-like pigmentation; SDF-1 identified as a key regulator of UV-induced hyperpigmentation	[108]
				Park *et al.*	2022	*Ex vivo*	SL lesional skin	Clearance of senescent cells significantly reduces pigmentation	[109]
				Murase *et al.*	2020	*ex vivo*	SL lesional skin tissue	Restoration of autophagy via mTORC1 inhibition significantly improves pigmentation and epidermal differentiation	[107]
				Yoon *et al.*	2018	2D co-culture + *ex vivo*	Fibroblast–melanocyte co-culture; normal human skin biopsies	Senescent fibroblasts increase melanin content and tyrosinase activity; pigmentation enhancement validated at tissue level	[102]
PIH	Post-inflammatory or injury states (acne, eczema, trauma, procedures); more frequent and persistent in darker skin types	Inflammatory mediators activate MITF-driven melanogenesis and transfer. Epidermal–dermal junction disruption enables dermal melanin deposition and macrophage uptake, sustaining deeper pigmentation.	Melanocytes; keratinocytes; immune cells; dermal macrophages; epidermal–dermal junction	Branquinho *et al.*	2020	2D + 3D	Melanocytes, keratinocytes, fibroblasts	L-tyrosine + NH₄Cl induce melanogenesis; basal-layer melanin deposition confirmed histologically in 3D models	[101]
				Zhao *et al.*	2022	2D	HaCaT keratinocytes, human epidermal melanocytes	Separate UVB irradiation of keratinocytes and melanocytes to model inflammatory and melanogenic pathways	[103]
Melasma	UV exposure; hormonal factors; genetic predisposition	Paracrine factors sustain melanocyte hyperactivation, while basement membrane alteration and dermal remodeling promote pigment retention and lesion stability.	Melanocytes; keratinocytes; fibroblasts; endothelial cells; epidermal–dermal junction	Alcantara *et al.*	2020	*Ex vivo*	Facial melasma lesion and adjacent uninvolved skin explants	UVB increases basal-layer melanin density; distinct response patterns between lesional and adjacent skin	[98]
				Byun *et al.*	2016	2D + 3D	Lesional/perilesional fibroblasts, human epidermal melanocytes; pigmented epidermal equivalents	Lesion-associated fibroblasts enhance melanogenesis	[82]
Vitiligo	Genetic susceptibility; oxidative stress/environmental stressors	Melanocyte oxidative stress activates innate immunity and Th1 responses. CD8⁺ T cells produce IFN-γ and recruit inflammation via the CXCL9/10–CXCR3 axis, inducing melanocyte apoptosis, detachment and exhaustion.	Melanocytes; keratinocytes; CD8⁺ T cells; epidermal–dermal junction	Komatsu *et al.*	2025	2D co-culture + 3D	iPSC-derived melanocytes, keratinocytes, human 3D skin model (T-Skin™)	IFN-γ induces dose-dependent reduction in melanocyte survival and dendritic architecture	[92]
				Boukhedouni *et al.*	2020	2D co-culture + 3D skin model + *ex vivo*	Melanocytes, keratinocytes; keratinocytes–melanocytes co-culture; vitiligo patient skin	TNF-α/IFN-γ increase MMP-9 and disrupt E-cadherin, causing melanocyte instability/detachment; JAK1/3 inhibitor (tofacitinib) reduces detachment via MMP-9 suppression	[93]
				Regazzetti *et al.*	2015	*Ex vivo*	Vitiligo patient-derived skin explants	Oxidative stress–induced suppression of WNT signaling; WNT agonists drive residual melanocyte stem/progenitor cells toward premelanocyte differentiation	[95]
				Kumar *et al.*	2012	2D co-culture	Keratinocytes, melanocytes (from vitiligo patients/controls)	Intrinsic phenotypic differences in patient-derived cells, including altered morphology, adhesion and pigment maintenance capacity	[91]
				van den Boorn *et al.*	2009	*Ex vivo*	Autologous normally pigmented skin explants, T cells from vitiligo lesion margins	T-cell infiltration and melanocyte killing	[94]
				Mosenson *et al.*	2013	*Ex vivo*	Vitiligo patient-derived skin explants	Immunomodulatory intervention validated: inducible HSP70 (HSP70i) suppresses T-cell-mediated depigmentation	[96]

SL, solar lentigo; PIH, post-inflammatory hyperpigmentation; iPSC, induced pluripotent stem cell; SASP, senescence-associated secretory phenotype; TYR, tyrosinase; ET-1, endothelin-1; UV, ultraviolet; mTORC1, mechanistic target of rapamycin complex 1; IFN-γ, interferon gamma; TNF-α, tumor necrosis factor alpha; MITF, microphthalmia-associated transcription factor; CXCL, C-X-C motif chemokine ligand; CXCR3, C-X-C motif chemokine receptor 3; Th1, type 1 helper T cell; MMP-9, matrix metalloproteinase 9.

## Challenges and future perspectives

Current *in vitro* model systems for studying pigmentary disorders have evolved from 2D cultures to multi-level platforms, including reconstructed epidermis or full-thickness skin models, organoids and skin-on-a-chip systems. However, substantial inter-laboratory variability in cell sources, culture conditions, stimulation protocols and evaluation methods continues to limit model reproducibility and cross-study comparability [58]. At the biological level, existing models can adequately recapitulate fundamental processes such as melanogenesis and melanosome transfer, but their ability to simulate key pathological mechanisms involved in chronic and recurrent pigmentary disorders, such as dermal microenvironment remodeling, sustained immune cell activation and regulation by vascular and neural signaling in conditions such as melasma and PIH, remains limited [90]. Therefore, future model development should move beyond reproducing pigmentation phenotypes and focus on reconstructing the pathological processes responsible for disease initiation, progression and persistence.

From the perspective of functional assessment and readouts, conventional *in vitro* pigmentation models continue to rely predominantly on endpoint analyses, including total melanin content, tyrosinase activity or quantitative immunostaining, which are insufficient to capture the dynamic processes of melanin synthesis, transport and deposition. By contrast, constructing full-thickness skin models incorporating melanocytes and other functional cell types on organ-on-a-chip platforms, maintaining tissue maturity via ALI and continuous perfusion and integrating online, noninvasive and continuous monitoring approaches such as TEER measurements may more closely mimic *in vivo* physiological and pathological environments and substantially enhance model detectability and predictive capacity [84]. For these platforms to function as NAMs, evaluation should move from descriptive resemblance to predictive validity: clinically relevant directionality, dose-response behavior, temporal dynamics and known therapeutic or adverse-response patterns should be benchmarked against human tissue, patient-derived data or clinical observations [88, 89]. In pigmentary disorders, useful benchmark perturbagens may include UV or visible light exposure, IFN-γ/TNF-α stimulation, ET-1 or α-MSH induction, oxidative stress, senescence-associated stimulation and reference depigmenting or repigmenting agents.

Looking ahead, melanocyte-containing skin models should advance along four linked directions. First, disease-oriented multimodular integration should incorporate immune cells, perfusable vascular networks, neural components and disease-relevant dermal microenvironments into skin equivalents or skin-on-a-chip systems. Such integration is particularly important for modeling immune-mediated depigmentation, chronic inflammatory hyperpigmentation and senescence-associated pigmentary disorders, in which melanocyte behavior is regulated by complex interactions among epidermal, dermal, vascular and immune compartments. Recent multimodal multiorgan-on-a-chip platforms, although not specific to pigmentary disorders, illustrate how dynamic perfusion, organ–organ coupling and integrated sensing strategies can be combined to study tissue interactions and drug metabolism [90, 110]. Second, programmable and personalized construction should integrate iPSC technologies, gene editing and 3D bioprinting to generate models with patient-specific pigmentation backgrounds, genetic susceptibility and disease-associated cellular phenotypes. Such approaches may be particularly valuable for heterogeneous pigmentary disorders, where donor variation and disease susceptibility substantially influence treatment responses [111]. Third, quantitative readouts should include spatial imaging, Fontana–Masson or bright-field melanin segmentation, longitudinal live imaging, transcriptomics, secretome/cytokine profiling, barrier/permeability readouts and sensor streams such as TEER, oxygen, pH and inflammatory mediators. AI-assisted image analysis can convert subjective pigmentation scoring into spatially resolved phenotypes, including melanin area fraction, color uniformity, dendrite morphology, melanosome-transfer patterns, epidermal thickness, immune-cell infiltration and longitudinal pigment kinetics. These multimodal features may ultimately support prediction of therapeutic response, repigmentation potential or hyperpigmentation risk rather than merely describing pigmentation changes [87]. Fourth, translation requires standardized analytical validity, biological validity and context-of-use validity. Minimal reporting should include donor metadata, pigmentation background, passage number, melanocyte-to-keratinocyte ratio, matrix composition and stiffness, ALI duration, perfusion parameters, stimulation protocol, batch size and predefined acceptance criteria for barrier integrity, stratification, viability and baseline pigmentation [88]. Multi-site ring studies (inter-laboratory validation studies) using shared reference compounds and harmonized image-analysis pipelines will be essential for regulatory acceptance and industrial reproducibility.

Overall, *in vitro* models of pigmentary skin disorders are evolving from simplified, melanocyte-centered systems toward more complex platforms that integrate multicellular interactions, microenvironmental regulation and dynamic monitoring capabilities. With continued refinement of model construction and evaluation within standardized and translation-oriented frameworks, these *in vitro* models are expected to become indispensable tools for elucidating disease mechanisms, screening therapeutic strategies and enabling more predictive, mechanism-based and patient-relevant evaluation of pigmentary interventions. Figure 6 outlines this integrated skin-on-a-chip outlook for dynamic disease modeling, drug evaluation and cosmetic testing.

**Figure 6:**
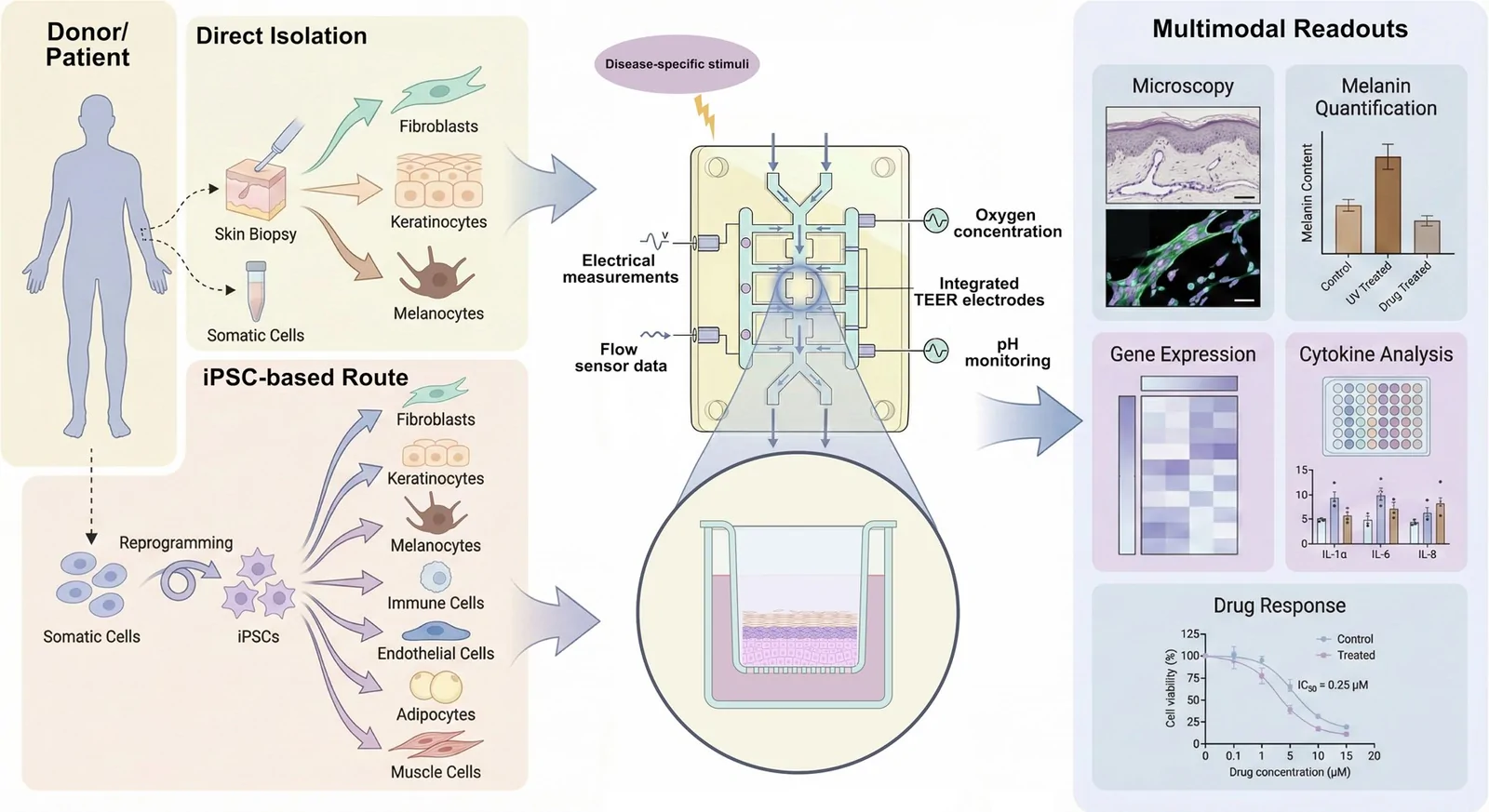
Conceptual outlook for pigmented skin-on-a-chip platforms. Primary or iPSC-derived cells can be organized into multicellular skin units containing melanocytes, keratinocytes, fibroblasts and immune or endothelial modules. Controlled perfusion, oxygen/pH regulation, TEER monitoring, imaging, cytokine analysis, gene-expression profiling and AI-assisted melanin quantification can support dynamic disease modeling, drug evaluation and cosmetic testing.
